# Biology and Model Predictions of the Dynamics and Heterogeneity of Chromatin-Nuclear Lamina Interactions

**DOI:** 10.3389/fcell.2022.913458

**Published:** 2022-05-26

**Authors:** Julia Madsen-Østerbye, Aurélie Bellanger, Natalia M. Galigniana, Philippe Collas

**Affiliations:** ^1^ Department of Molecular Medicine, Institute of Basic Medical Sciences, Faculty of Medicine, University of Oslo, Oslo, Norway; ^2^ Department of Immunology and Transfusion Medicine, Oslo University Hospital, Oslo, Norway

**Keywords:** chromatin, interaction, LAD, lamina-associated domain, nuclear envelope, polymer modeling, restraint

## Abstract

Associations of chromatin with the nuclear lamina, at the nuclear periphery, help shape the genome in 3 dimensions. The genomic landscape of lamina-associated domains (LADs) is well characterized, but much remains unknown on the physical and mechanistic properties of chromatin conformation at the nuclear lamina. Computational models of chromatin folding at, and interactions with, a surface representing the nuclear lamina are emerging in attempts to characterize these properties and predict chromatin behavior at the lamina in health and disease. Here, we highlight the heterogeneous nature of the nuclear lamina and LADs, outline the main 3-dimensional chromatin structural modeling methods, review applications of modeling chromatin-lamina interactions and discuss biological insights inferred from these models in normal and disease states. Lastly, we address perspectives on future developments in modeling chromatin interactions with the nuclear lamina.

## Introduction

The 3-dimensional (3D) conformation of the genome is critical for the orchestration of gene expression regulating development, cell differentiation and tissue homeostasis. Genome organization relies on chromosomal interactions (Rowley and Corces, 2018) and at the nuclear periphery, associations of chromatin with the nuclear lamina (NL) *via* lamina-associated domains (LADs) (Briand and Collas, 2020). Some LADs change during differentiation or are altered in disease, and laminopathies, pathologies caused by mutations in nuclear lamins (Shin and Worman, 2022), underscore the importance of maintaining a proper radial genome organization. Whereas the genomic landscape of LADs is getting well characterized, surprisingly little is known on how LADs are physically and mechanistically repositioned in the genome.

Computational modeling of chromatin structure (Parmar et al., 2019; Jerkovic and Cavalli, 2021) creates opportunities to better understand the patterns, dynamics and mechanisms of chromatin-NL interactions in normal and disease states. Polymer physics modeling provides quantitative information on the physical properties of chromatin folding. In addition, restraint-based methods model 3D chromatin structures represented by points and restraints between them dictated by wet-lab data. Both approaches can accommodate positional constraints for chromatin, for example imposing interactions between similar chromatin domains or interactions with a nuclear body or with a surface representing a NL.

Here, we highlight the heterogeneous nature of the NL and LADs, outline the main 3D chromatin structural modeling methods currently used, review computational models of chromatin-NL interactions, and discuss biological insights deducted from these models in normal and pathological conditions. Lastly, we address perspectives on applications of modeling interactions of chromatin with the NL with the aim of better appreciating the multiple facets of functional genome organization.

## Heterogeneity of the Nuclear Lamina and Lamina-Associated Domains

Current views of 3D nuclear architecture depict a hierarchical and dynamic environment where chromatin can alter its composition and conformation in response to stimuli (Rowley and Corces, 2018). Within chromosome territories, chromatin is divided into active and inactive compartments, within which smaller topological domains reflect a high frequency of chromosomal contacts thought to regulate gene expression. These topological domains can also form dynamic long-range interactions within chromosomes, while some also interact with the NL *via* LADs, and thereby radially organize the genome (Marti-Renom et al., 2018; Buchwalter et al., 2019).

### The Heterogeneous Nuclear Lamina

At the nuclear periphery, the NL interfaces the inner nuclear membrane and chromatin as a meshwork of intermediate filaments built from polymers of A-type lamins (lamins A and C, splice variants of the *LMNA* gene) and B-type lamins (lamins B1 and B2, products of the *LMNB1* and *LMNB2* genes) (Burke and Stewart, 2013). The NL plays a critical role in maintaining nuclear shape. It provides mechanical support to chromatin and anchors chromatin modifying enzymes, transcription factors and signaling molecules, imposing a spatio-temporal regulation of genome compaction, DNA replication and transcription (Buchwalter et al., 2019). Studies combining cryo-electron tomography and microscopy reveal that the NL forms a heterogeneous structure with distinct but interacting networks of A- and B-type lamin homopolymers and void space occupied by other proteins and chromatin (Shimi et al., 2008; Shimi et al., 2015; Turgay et al., 2017). Additional imaging data show that lamin B1 and lamin A/C form concentric but overlapping networks with lamin B1 localized more outwards, adjacent to the inner nuclear membrane (Nmezi et al., 2019). Interestingly, models of NL structure inferred from these findings have been shown to predict NL behavior, the roles of lamin B1 and A/C networks and impacts of their perturbation on nuclear function (Nmezi et al., 2019). Whether this structural organization of the NL provides a basis for differential interactions of A- and B-type lamins with chromatin (Forsberg et al., 2019) remains unknown but is a possibility. As addressed later in this review, these observations bring about options to enhance the prediction potential of 3D models of chromatin folding and interactions with the NL. Computational models of the relationship between components of the nuclear envelope have been discussed elsewhere (Nmezi et al., 2019; Sapra et al., 2020; Tenga and Medalia, 2020; Kittisopikul et al., 2021) and provide complementary insights to those highlighted here on the structural organization of the periphery of the mammalian nucleus.

### Lamina-Associated Domains Are Diverse and Dynamic Genome Organizers

**FIGURE 1:**
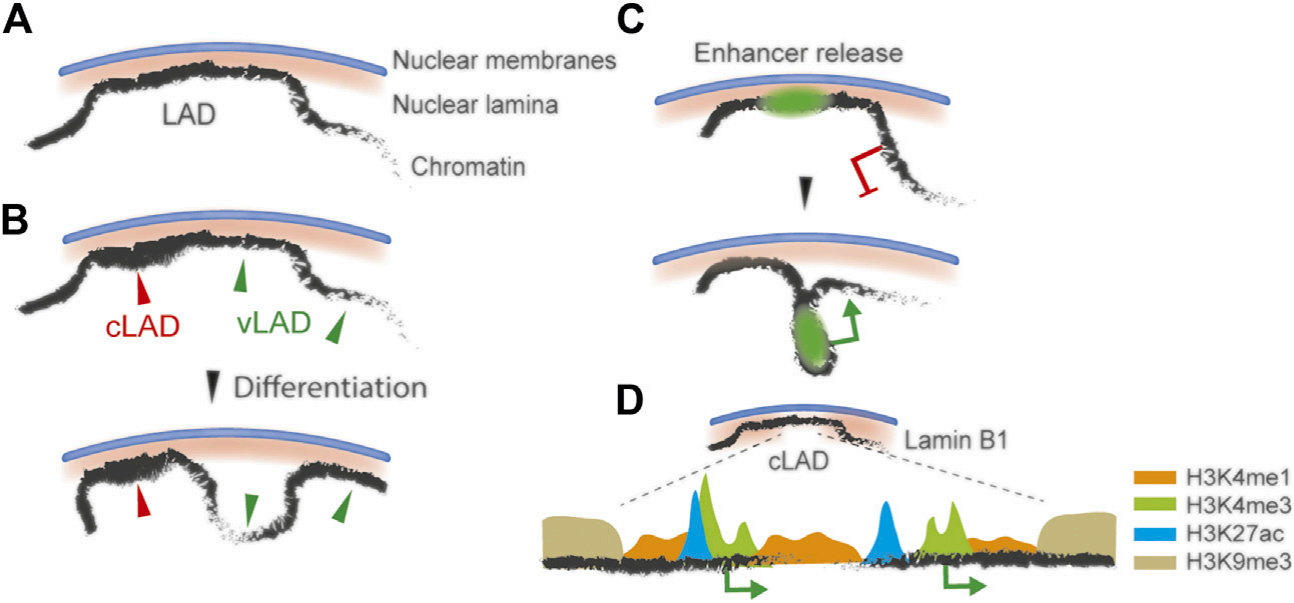
Dynamics and heterogeneity of LADs **(A)** Interaction of chromatin with the NL *via* a LAD **(B)** Variable LADs (vLADs) are repositioned in the genome during differentiation **(C)** Enhancer (green area) release from the NL by LAD detachment, and activation of a nearby gene (green arrow) **(D)** Active cLAD sub-domain of lower lamin B1 level than the rest of the LAD, depleted of H3K9me3 but enriched in euchromatic histone modifications (H3K4me1, H3K4me3 and H3K27ac). Genes in these sub-domains escape the heterochromatic repressive environment of LADs.

In mammalian cells, hundreds of LADs have been mapped throughout the genome (Figure 1A) using various wet-lab and bioinformatics methods (Briand and Collas, 2020; Manzo et al., 2022). Irrespective of these methods, LADs have been identified as domains of about 10 kilobases (kb) to 10 megabases (mb) unevenly distributed between chromosomes and within chromosomes. LADs are AT-rich and of low gene density, and enriched in long interspersed nuclear elements (LINEs) and features of heterochromatin such as histone H3 lysine 9 dimethylation (H3K9me2) and H3K9me3 (Guelen et al., 2008). LADs tend to display relatively sharp borders (sharp transitions between LAD and non-LAD regions) and are typically flanked by active genes, and within 50–200 kb of these borders, by domains of H3K27me3 (Harr et al., 2015; Paulsen et al., 2019). As a result, most genes in LADs are silent or expressed at low levels and overall, LADs form repressive domains at the nuclear periphery. Likely as a result of their compact state, LADs are excluded sites of viral (e.g., HIV-1) integration despite preferred virus insertions at the nuclear periphery (Marini et al., 2015), and constitute domains of low DNA lesion repair capacity presumably due to limited access to the DNA repair machinery (Garcia-Nieto et al., 2017).

LADs are a general feature of genome organization, but not all LADs have similar properties (Figure 1B). Some are well conserved between cell types (Keough et al., 2020; Meuleman et al., 2013), during differentiation (Madsen-Østerbye et al., 2022; Peric-Hupkes et al., 2010; Rønningen et al., 2015) and across circadian time (Brunet et al., 2019). These constitutive LADs (cLADs) are characterized by high lamin B1-chromatin contact frequency and are viewed as the genomic backbone of chromosome anchoring to the nuclear envelope. Other LADs are less conserved between cell types (Figure 1B). Variable (v)LADs are smaller than cLADs, display lower lamin B1 enrichment, harbor a higher gene density and are less heterochromatic. vLADs are a feature of differentiation where entire LADs or LAD edges associate with or detach from the NL (Madsen-Østerbye et al., 2022). Some loci in vLADs are repositioned away from the NL when they lose lamin association (Reddy et al., 2008), but this is not systematic (Robson et al., 2016; Forsberg et al., 2019; Czapiewski et al., 2022). LAD repositioning also occasionally concurs with activation of cell type-specific genes (Peric-Hupkes et al., 2010; Robson et al., 2016; Keough et al., 2020; Czapiewski et al., 2022; Madsen-Østerbye et al., 2022) or disease-specific genes (Kohler et al., 2020). vLADs may also function as regulators of transcription by releasing enhancers that in turn regulate expression of neighboring genes (Robson et al., 2016; Czapiewski et al., 2022) (Figure 1C).

In spite of their overall conservation, increasing evidence indicates that cLADs are not homogeneous in their chromatin composition. Approximately 10% of genes in cLADs have initially been found to be expressed and to escape the repressive NL environment (Guelen et al., 2008), a figure which has been confirmed in many studies regardless of cell type and species. Lower local lamin B1 enrichment, promoter sequence properties and active histone modifications may account for this apparent discrepancy (Brueckner et al., 2020; Leemans et al., 2019; Madsen-Østerbye et al., 2022; Wu and Yao, 2017) (Figure 1D). These regions also appear to be more prone to alterations in epigenetic states and chromatin accessibility than the more constitutive heterochromatic domains of LADs, For example, in diseases caused by lamin mutations such as Hutchinson-Gilford Progeria Syndrome (HGPS), a premature aging laminopathy caused by mutations in the *LMNA* gene (Kohler et al., 2020; Shin and Worman, 2022).

Additionally, a subset of LADs bound by A-type lamins harbors features of euchromatin (Lund et al., 2015; Gesson et al., 2016), and lamin C, when phosphorylated, can bind H3K27-acetylated enhancers (Ikegami et al., 2020). A fraction of B-type lamins also intriguingly binds active genes during the epithelial-to-mesenchymal transition (Pascual-Reguant et al., 2018), through currently unidentified mechanisms. This variation of chromatin states in LADs creates opportunities to better appreciate the physics of chromatin-NL interactions and tentatively predict their functional implications.

## 3D Models of Chromosomes Provide Mechanistic and Statistical Insights Into Chromatin Dynamics

One strategy to investigate spatial genome dynamics is to generate 3D models of chromatin and analyze properties of the models. Modeling enables statistical and mechanistic insights into principles of chromatin folding or interaction with a surface representing, for example, the NL. Models of chromatin have been generated using two main approaches: 1) polymer physics models chromatin as a semi-flexible polymer chain that can adopt many configurations within physical constraints applied to the chain; 2) restraint-based modeling represents chromatin by beads in a Euclidian space with restrained interactions between them commonly determined from chromosome interaction data. We next provide an account of these modeling approaches in light of their relevance for modeling chromatin configuration at the NL. For details on 3D genome modeling methods, we refer to an excellent exhaustive review (Jerkovic and Cavalli, 2021).

### Polymer Models of Chromosomes

Polymer modeling provides quantitative information on the physical properties of chromatin folding and on chromosome dynamics in the nucleus (Fiorillo et al., 2019; Tortora et al., 2020). Polymer models can predict statistical quantities such as end-to-end (Euclidian) distances or interaction frequencies between monomers in the polymer. A chromosome or chromosome segment is typically modeled as a semi-flexible polymer chain (Parmar et al., 2019). Semi-flexible polymers can in principle adopt an infinite number of configurations, but these are in reality limited by the persistence length L_P_ of the polymer—that is, the length under which the polymer behaves as a rigid rod and above which it behaves as a flexible chain. The repeating units of chromatin, modeled as monomers in the polymer chain, further limit the number of conformations the polymer can adopt during simulations; this limitation is typically achieved by introducing a self-avoidance effect to prevent clashes between monomers (Chiariello et al., 2016).

**FIGURE 2:**
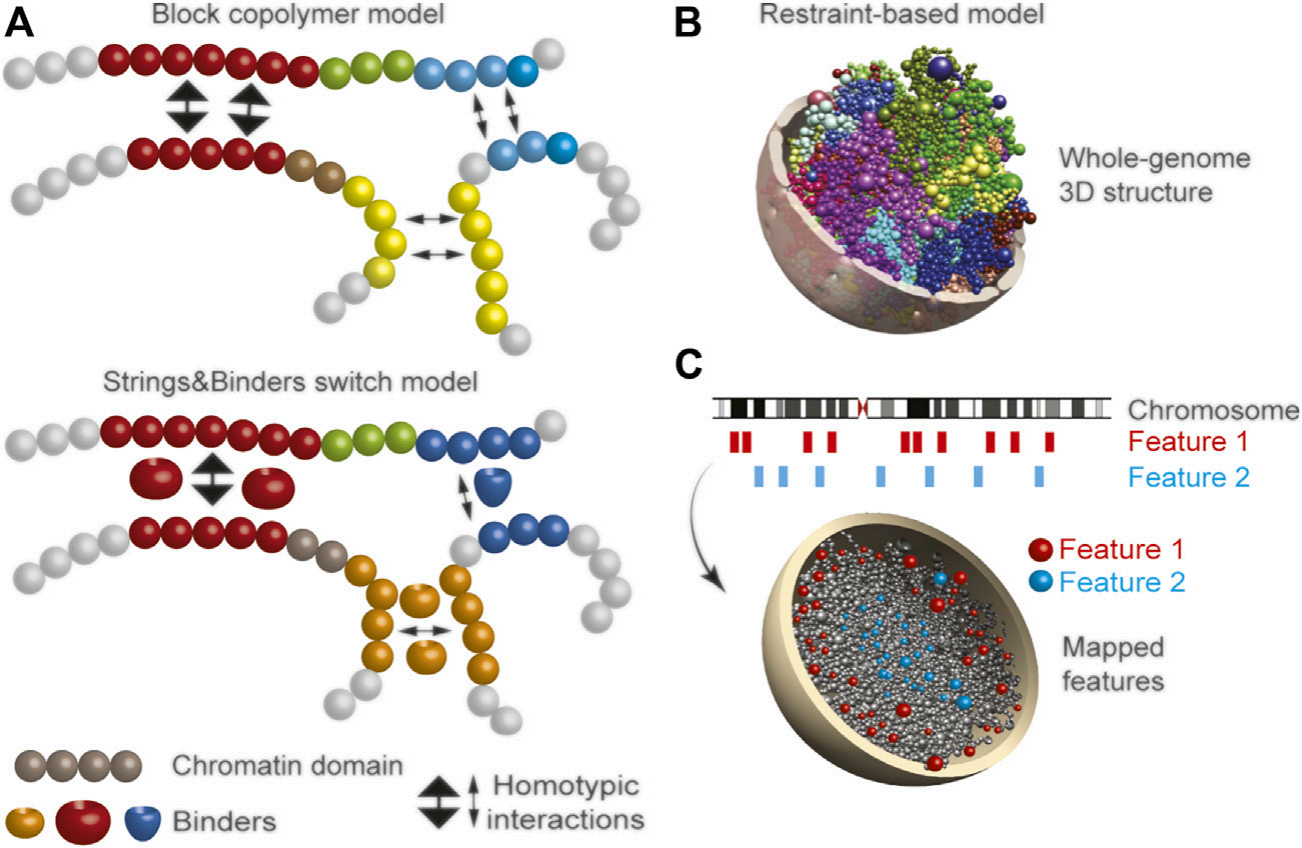
Main polymer physics modeling approaches for chromatin **(A)** Top, block copolymer modeling of chromatin folding relying on homotypic interactions between domains (blocks) of similar epigenomic signatures (colors). Variations the strength of these interactions are introduced to model heterochromatic (thick arrows) or euchromatic (thin arrows) homotypic interactions. Bottom, a variant of block copolymer modeling: here, “binders” (e.g., transcription factors) mediate homotypic chromatin interactions **(B,C)** Restraint-based modeling **(B)** 3D model example of a whole human genome structure; each color defines a chromosome as a chain of beads, with one bead representing a topological domain identified from Hi-C data (here, in adipose stem cells). The model integrates Hi-C and lamin B1 ChIP-seq (LAD) restraints for chromatin and is generated with our Chrom3D platform (Paulsen et al., 2017) **(C)** 3D chromatin modeling enables spatial visualization of genomic features not detectable in 1D data; here, feature 1 is more peripherally located than feature 2.

Block copolymer modeling is a broadly used generic and minimal chromatin modeling technique. It operates on the assumption that chromatin is a self-avoiding polymer whose folding is dictated by preferential interactions between domains (blocks) of similar epigenomic signatures, or “colors” (Jost et al., 2014) (Figure 2A). Self-avoiding consecutive monomers (beads) are connected *via* a harmonic potential and an interaction strength between beads can be added *via* a tuneable attraction potential: for example, a strong potential can model homotypic heterochromatic interactions reflecting a compact structure, while a weaker potential models homotypic euchromatic interactions reflecting the more open state of active chromatin (Jost et al., 2014). Despite their simplicity and the exclusion of biological aspects of chromatin folding, block co-polymer models can recapitulate large scale Hi-C contact maps including A/B compartments and topological domains when built from epigenomes (Jost et al., 2014).

A variation of block copolymer modeling referred to as Strings & Binders switch modeling, allows interaction of chromatin with factors (binders) that mediate these interactions (Annunziatella et al., 2018; Barbieri et al., 2012) (Figure 2A). Again, beads are given a chromatin state (color) based on the type of binder they are attracted to, such as architectural proteins (e.g., HP1α/CBX5, CTCF), histone-modifying enzymes (e.g., histone deacetylase HDAC3) or transcription factors (e.g., SREBP1). Beads tend to interact with binders and other beads of the same type as binder concentration increases, forming homotypic interactions. These models can be initiated from contact matrices by applying polymer physics laws, or by applying *a priori* knowledge of the modeled chromatin region, such as chromatin states or transcription factor binding profiles. Block copolymer simulations can also model the behavior of a chromatin chain that can assume various thicknesses and compositions, providing a more realistic view of chromatin (Buckle et al., 2018). Polymer models have mainly been limited to intra-chromosomal interactions, but with modifications, can also model interactions between multiple chromosomes (Oliveira Junior et al., 2021).

### Restraint-Based Modeling of Chromatin

Other chromatin 3D modeling approaches use restraint-based methods to infer spatial information directly from experimental data and reconstruct structures without assumptions on folding mechanisms. Contact matrices derived from Hi-C other 3C-sequencing data are used to identify pairs of interacting domains as primary constraints. One restraint-based approach is to reconstruct a single consensus structure representing the average of 3D conformations in the cell population under study (Duan et al., 2010; Hu et al., 2013; Zhang et al., 2013; Lesne et al., 2014; Szalaj et al., 2016; Zou et al., 2016; Wlasnowolski et al., 2020). Consensus structures provide insights into genome architecture, but by definition do not capture variations in chromatin conformation seen between cells in a population (Nagano et al., 2013; Cheng et al., 2020).

To enable this, other methods simulate many structures. Resampling methods carry out a large number of independent optimizations from the same input data. Optimizations start from, most commonly, a random chromosome configuration and use the same scoring function aiming to reach a state where no constraint violations remain, producing a quasi-stable structure (Bau and Marti-Renom, 2011; Di Stefano et al., 2016; Kalhor et al., 2012; Le Dily et al., 2014; Meluzzi and Arya, 2013; Paulsen et al., 2017; Tjong et al., 2012) (Figure 2B). Of note, optimization can also be initialized from a determined (phenomenological) chromosome disposition based on existing data, for example, describing the radial positioning of chromosomes in the nucleus (Di Stefano et al., 2016). Other methods deconvolute Hi-C data into a population of 3D structures using various techniques (Tjong et al., 2016; Li et al., 2017; Zhu et al., 2017). To enhance accuracy of the models, chromatin constraints can be added to for instance prevent clash between beads (motivated by chromatin thickness), position beads towards a nucleolus, or direct them towards a NL (Duan et al., 2010; Di Stefano et al., 2016; Li et al., 2017; Paulsen et al., 2017; Pouokam et al., 2019). Hi-C-constrained models of the diploid human genome have been shown to recapitulate features of spatial genome organization, including associations with a nuclear envelope (by including LAD constraints), with functional relevance (Di Stefano et al., 2016).

We have also reported ensembles of chromatin structures (Paulsen et al., 2017) relying on Hi-C and lamin B1 ChIP-seq (LAD) data, which faithfully recover characteristics of radial genome organization and stability observed in single cells (Kind et al., 2015). The structures allow inference on the regionalization of chromatin states (Di Stefano et al., 2016; Paulsen et al., 2018; Paulsen et al., 2019), and on radial positioning of loci (Briand et al., 2018), disease-associated LADs (Paulsen et al., 2017) and cancer mutations (Garcia-Nieto et al., 2017) (Figure 2C). It will be interesting to compare outputs of restraint-based model ensembles and of models generated from single-cell data (Cardozo Gizzi, 2021; Kos et al., 2021) to determine the most powerful strategy for predicting chromatin structure dynamics. This would be relevant in the study of 3D cancer genomes, as cell-to-cell heterogeneity within tumors hampers many investigations.

## Modeling Interactions of Chromatin With the Nuclear Lamina

Chromatin folding at, and interactions with, the NL have been modeled in attempts to identify physical processes driving these events, infer mechanisms of chromatin association with, and dissociation from, the NL, and provide more accurate spatial genome structures at the nucleus level.

### Basic Physical Considerations in Modeling Interactions of a Chromatin Polymer With a NL Surface

**FIGURE 3:**
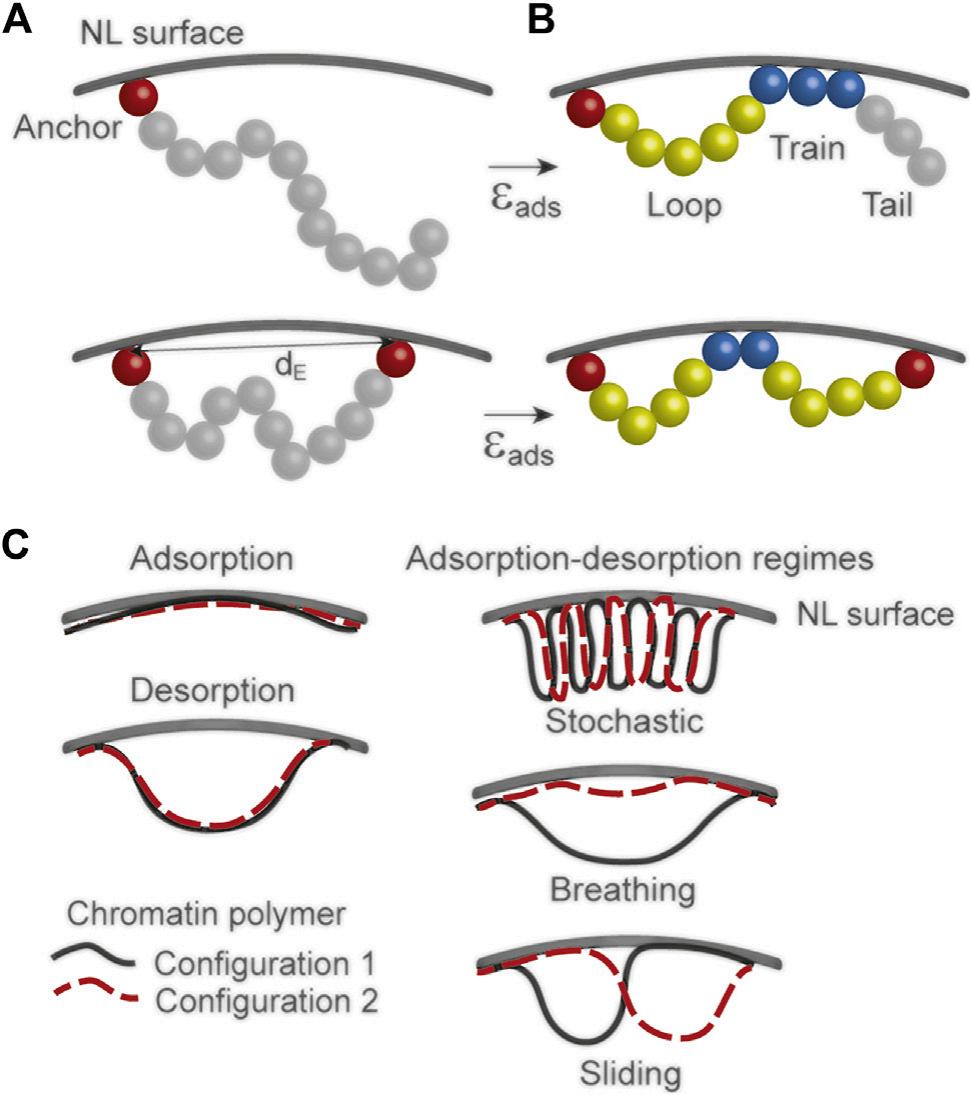
Polymer modeling of chromatin interactions with a NL surface **(A)** Chromatin is modeled as a chain of beads pinned to a hard surface by one or two anchors (red beads) separated by a Euclidian distance d_E_. This distance can be tuned at the start of simulations to generate a loose or stretched polymer chain near the surface **(B)** During simulations, the polymer changes conformation near the surface. Applying an attraction potential (ε_ads_) from the surface elicits loop, train and tail polymer configurations whose position, size and frequency vary by tuning ε_ads_ and physical parameters of the polymer (e.g., stiffness, contour length and d_E_) **(C)** Interpretations of a chromatin polymer behavior at a NL surface, inferred from polymer configurations. Variations in ε_ads_ from the surface leads to full adsorption, full desorption or various adsorption-desorption regimes; adapted from our own work (Brunet et al., 2021).

We have recently assessed the extent to which basic physical properties of a polymer, such as stiffness and stretching, would influence its configurations near an impermeable (hard) surface representing a NL, fitted with an attraction potential towards the polymer (Brunet et al., 2021). Chromatin is modeled as a polymer of hard beads of contour length L_C_ 360 nm representing a ∼50 kb region to enable modeling interactions of small vLADs or euchromatic sub-LAD regions (Madsen-Østerbye et al., 2022). The polymer is configured with one or two ends anchored to the surface with increasing Euclidean distance d_E_ between them, yielding a relaxed or stretched chain (Figure 3A). Further, by varying the persistence length L_P_, or stiffness, of the polymer, the behavior of euchromatin (low persistence length) or heterochromatin (higher persistence length) at the NL can be approximated (Brunet et al., 2021).

Data from simulations indicate that in the absence of attraction potential from the NL surface, the polymer must be pinned by at least one bead to enable any interactions with the surface. Second, a flexible chain explores a greater area than a semi-flexible or rigid chain, so one may infer that a euchromatic region can explore a greater space near the NL than a more rigid heterochromatic domain (Brunet et al., 2021). Third, interaction profiles of the polymer with the surface, described as trains, tails and loops (Figure 3B) during simulations suggest that more flexible (eu)chromatin as opposed to more rigid (hetero)chromatin can adopt more variable and dynamic configurations at the NL, reflecting biological observations (Madsen-Østerbye et al., 2022). It will be informative to investigate the functional impact of this property of euchromatic LADs (Pascual-Reguant et al., 2018; Ikegami et al., 2020; Liu and Ikegami, 2020) or sub-LAD domains (Madsen-Østerbye et al., 2022) on gene expression dynamics in these regions.

Without attraction potential, polymer interactions with the surface are only transient and barely detectable (except at the anchors). This is consistent with other models where turning off an attraction potential between the NL surface and a chromatin polymer promotes polymer detachment (Chiang et al., 2019; Falk et al., 2019; Sati et al., 2020; Amiad-Pavlov et al., 2021). In contrast, applying an attraction potential to the NL is essential for persistent interactions. Tuning this potential yields interaction profiles ranging from stable adsorption (strong potential) to stable desorption (weak potential), with in between, adsorption-desorption regimes yielding multiple configurations at the surface (Brunet et al., 2021) (Figure 3C). These experiments together identify fundamental physical parameters which in combination contribute to predict chromatin behavior at the NL.

The absence of attraction potential towards a NL may still enable expected polymer positioning relative to a physical constraint. Indeed, when a self-avoiding polymer is confined to a nucleus sphere, non-specific entropic forces alone can remarkably shape and position chromatin polymers in the sphere and approximate high-order localization of loose (thin) and compact (thick) segments in the sphere center or periphery, respectively (Cook and Marenduzzo, 2009).

### Modeling Nucleus-Wide Chromatin Reconfiguration With a Minimal Set of Physical Parameters

These findings have been extended by modifying the polymer-surface interaction parameter as a fraction of the chromatin polymer bound to the NL, and introducing a chromatin volume fraction (modeling a hydration effect) and an intra-chromatin attraction potential (Bajpai et al., 2021). Tuning these parameters in simulations of chromatin behavior in a nucleus sphere yields transitions in chromatin reconfigurations, from peripheral heterochromatin enrichment to a fully central “collapsed” localization when the LAD parameter is turned off. The data reveal that a theoretical competition between chromatin-NL and chromatin-chromatin attraction strengths is sufficient to determine large-scale chromatin arrangement in the nucleus (Bajpai et al., 2021).

Identification of a minimalistic set of parameters able to predict chromatin conformation is useful (Bajpai et al., 2021); however the models would gain from the inclusion of attraction potentials regulating homotypic and heterotypic chromatin interactions. For instance, abrogating chromatin-NL interactions should not result in chromatin clumping in the nucleus center. Rather, turning off chromatin-NL interactions has been shown to reconstitute the central localization of heterochromatin observed in “inverted” nuclei, with a peripheral localization of euchromatin (Falk et al., 2019) (see also below). Similarly, turning off the LAD parameter in restraint-based genome models results in less stable peripheral localization of peripheral chromosomes across simulations, but chromatin does not collapse in the nucleus center despite persistent interactions between topological domains (Paulsen et al., 2017). That said, the models of Bajpai et al. seem to recapitulate *in vivo* chromatin imaging observations and, with only a small number of parameters, account for changes in phase separation (chromatin vs. aqueous) that may drive mesoscale chromatin reconfiguration in developing *Drosophila* larvae (Amiad-Pavlov et al., 2021).

## When Modeling Meets Biology

### Polymer Models Predict That Attachment to the NL Compacts Chromatin

**FIGURE 4:**
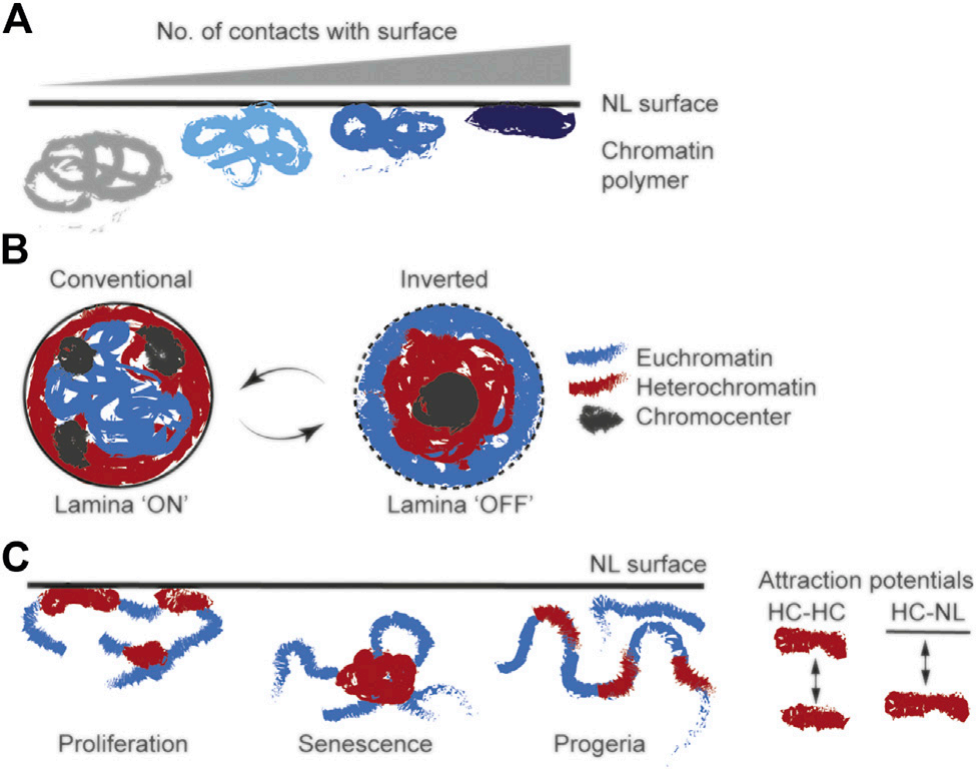
Chromatin configurations at a NL predicted from polymer models near a hard surface **(A)** Conformation of a chromatin polymer fitted with an increasing number of contact points with a NL surface in simulations (Ulianov et al., 2019): increasing the number of contacts decreases LAD volume and ultimately results in a “pancake” configuration at the surface **(B)** Nucleus-wide radial arrangement of heterochromatin and euchromatin compartments in models of conventional and inverted nuclei; turning off a NL attraction potential (lamina OFF) results in the inverted configuration with euchromatin at the nuclear periphery (Falk et al., 2019) **(C)** Chromatin configuration at a NL surface after modeling cells in proliferating, senescence and progeroid states; variations in configurations result from tuning attraction potentials between heterochromatin (HC) blocks and between heterochromatin (HC) and NL (Chiang et al., 2019; Sati et al., 2020).

Polymer simulations of *Drosophila* S2 cell chromatin shows that interactions with a surface are sufficient to compact chromatin, with the degree of compaction being proportional to the number of contact points, ultimately reaching a “pancake” configuration (Ulianov et al., 2019) (Figure 4A). This is consistent with our theoretical findings from simulations of the dynamics of polymer adsorption to a surface (Brunet et al., 2021). Conversely, the models predict that release of LADs from the NL coincides with local decompaction of LAD chromatin, which was confirmed by microscopy (Ulianov et al., 2019). However, this does not imply that chromatin decompaction is a nucleus-wide phenomenon, because non-LAD domains undergo compaction upon LAD release from the NL (Sawh et al., 2020), presumably as a result of tension release in chromatin (Ulianov et al., 2019; Sawh et al., 2020).

### Polymer Models of Nuclear Inversion Uncouple Chromatin Compartmentalization From NL Interaction in the Radial Arrangement of the Genome

Block copolymer models have been used to disentangle the role of hetero-/euchromatin compartmentalization and of the NL in the radial disposition of chromatin in conventional vs. inverted nuclei in which heterochromatin is concentrated in the center while euchromatin lies towards the periphery (Falk et al., 2019). Simulations were done considering pericentric constitutive heterochromatic-, heterochromatic- and euchromatic-type monomers with or without interactions with a NL surface. These monomers were conferred with homotypic and heterotypic short-range interactions enabled by attraction potentials of various strengths. Remarkably, the models recapitulate genome compartmentalization seen in Hi-C data and produce the inverted radial chromatin conformation (Falk et al., 2019) reported in nuclei in the absence of lamin A or of the lamin B receptor (LBR) (Solovei et al., 2013) (Figure 4B). Only when short-range attraction potentials between heterochromatin monomers and the NL surface are introduced in simulations do the models adopt a conventional nucleus configuration with heterochromatin at the periphery (Falk et al., 2019); this is notably reversible (Figure 4B). Supporting original microscopy observations (Solovei et al., 2013), these models suggest that homotypic heterochromatic interactions are sufficient to drive the segregation of heterochromatin from euchromatin, whereas interaction with the NL is necessary to confer a conventional radial nuclear configuration (Falk et al., 2019).

### Modeling Chromatin Rearrangement and NL Dissociation During Senescence

Hallmarks of senescence are the formation of senescence-associated heterochromatic foci (SAHF) and degradation of the NL, which releases heterochromatin from the nuclear envelope and elicits SAHFs (Sadaie et al., 2013; Shah et al., 2013). To disentangle heterochromatin compaction from LAD repositioning in SAHF formation, block copolymer models have been generated, with tuneable attraction potentials between monomers in a chain modeling a senescence-associated heterochromatin domain, and between monomers and a NL surface (Sati et al., 2020) (Figure 4C). Modeling proliferating cells produces structures where heterochromatin masses interact but also occur away from the NL (Figure 4C). Moreover, a “membrane release” scenario where chromatin-NL attraction is brought to zero during simulations, effectively recapitulates LAD detachment from the NL. This results in local chromatin polymer decompaction and large-scale interactions consistent with SAHF formation away from the NL (Figure 4C). Tuning the attraction potential between monomers in the chromatin chain approximates imaging data (Sati et al., 2020), indicating that polymer models recapitulate both chromatin folding patterns and dynamic interactions with the NL.

Senescence-associated loss of heterochromatin at the NL also occurs after down-regulation of LBR, a protein of the inner nuclear membrane anchoring heterochromatin at the nuclear envelope (Herman et al., 2021). A role of LBR in mediating chromatin-NL interactions could be tested *in silico* by introducing LBR as a “binder” mediating these interactions in block copolymer models (see Figure 2A). Large-scale changes in nuclear morphology observed upon LBR down-regulation-elicited senescence (Kohler et al., 2020) would provide a valuable dataset to predict chromatin structural alterations at the nucleus level in restraint-based 3D genome models.

## Modeling Chromatin-NL Interactions to Better Understand Disease?

Disruption of chromatin architectural genomic elements or proteins has emerged as a mechanism underlying diseases ranging from developmental defects to laminopathies and certain cancers (Lupianez et al., 2016; Evangelisti et al., 2022; Shin and Worman, 2022). The discovery of nuclear architectural defects linked to these diseases provides opportunities to test whether modeling would help understand consequences of the diseases on genome integrity, and in the most optimistic scenarios help identify causes of the pathologies.

Polymer modeling of dynamic chromatin-NL interactions under normal and senescence conditions not only yields predictions on large-scale chromatin refolding upon detachment from the NL, but also extends our understanding of chromatin behavior at the NL in HGPS. By tuning only two attraction potentials controlling heterochromatin-heterochromatin and heterochromatin-NL interactions, simulations reproduce the distinct chromatin conformation changes occurring in senescent cells and in progeroid syndromes (Chiang et al., 2019) (Figure 4C). These simulations reveal euchromatic beads close to the NL despite the absence of specified attraction force between them, recapitulating the loss of peripheral heterochromatin reported in cells from HGPS patients (Shumaker et al., 2006; Kohler et al., 2020; Sebestyen et al., 2020).

Cell culture models of HGPS also provide opportunities to develop more elaborate and arguably more realistic models of chromatin changes in disease. Recent work highlights that alterations in chromatin accessibility, based on sequential extraction of chromatin fractions, in cells from HGPS patients can be measured in early passage HGPS cells prior to changes in heterochromatin composition (H3K9me3), which are only detectable in later passage (Sebestyen et al., 2020). Nonetheless, epigenetic remodeling by Polycomb (H3K27me3) seems to coincides with the structural changes of chromatin. These observations provide opportunities to temporally uncouple and mechanistically disentangle, in models of chromatin, the physical processes driving changes in chromatin structure and epigenomic changes, which most current models of chromatin assume are coincident. Because LADs are also monitored in the study (Sebestyen et al., 2020), temporal models of structural and biochemical alterations of chromatin in relation to the loss of association with the NL would also be plausible (Chiang et al., 2019).

### Explaining or Recapitulating Genome-Lamina Interactions With Modeling?

Data from simulations are not uncommonly interpreted as “explaining” a biological observation, as exemplified in some recent studies (Chiang et al., 2019; Sati et al., 2020). Whereas models may predict a biological outcome, they are a result of simulations carried out under minimalistic conditions and, we would argue, cannot per se explain biological phenomena. Results from simulation recapitulate a biological observation because parameters are appropriately tuned to mimic these observations. Notwithstanding, polymer models can generate useful working hypotheses on chromatin folding principles or mechanisms (defined by a minimal set of impactful parameters) underlying chromatin conformation and changes therein, e.g., in pathological contexts. Even based on simple rules or biophysical ingredients, models are believed to have the most useful if, on top of recapitulating biological observations, they can predict new ones. Models do not always need to be particularly sophisticated or detailed to achieve high predictive power, provided they capture sufficient details for the questions they aim to answer.

### Restraint-Based Models Enable New Hypotheses on Mechanisms Underlying a Pathology

Integration of genomic datasets from wet-lab experiments into restraint-based models of 3D genome structure have provided new spatial insights into genomic consequences of pathological states, which may open to new therapeutic avenues. For instance, statistical analyses of Chrom3D models of human fibroblast genomes indicate that UV-induced DNA lesions are predominantly detected in LADs, suggesting greater UV-susceptibility of chromosomes at the nuclear periphery (Garcia-Nieto et al., 2017). Even more relevant for cancer, nearly 80% of genes mutated in melanomas are not only found in LADs but also statistically enriched at the nuclear periphery in 3D genome models, while genes not mutated in melanomas are more centrally located (Garcia-Nieto et al., 2017).

Corroborating these findings, 3D genome models generated from Hi-C and radial positional information of loci reveal a decrease in the frequency of single nucleotide polymorphisms from the nuclear periphery towards the nucleus center, especially of those associated with melanomas or lung cancer (Girelli et al., 2020). This is again consistent with the higher frequency of cancer-linked mutations in late-replicating LAD heterochromatin (Schuster-Bockler and Lehner, 2012; Liu et al., 2013; Morganella et al., 2016). In contrast, loci implicated in gene fusions catalogued in The Cancer Genome Atlas are more centrally located than loci not involved in fusions; accordingly, the frequency of DNA double-strand breaks, which contribute the pathogenesis of gene fusions in cancers, also augments towards the nucleus center (Girelli et al., 2020). In another line of pathologies, altered lamin A/C-genome associations in nuclei from patients with a lipodystrophic laminopathy seem to occur preferentially in the nucleus center in 3D genome models of fibroblasts generated using public Hi-C data and control-vs. patient-specific lamin A/C ChIP-seq data (Paulsen et al., 2017).

These studies provide seminal examples of how restraint-based genome models may generate predictions on consequences of pathological insults or mutations on genome integrity with a 3D perspective. Currently, these models cannot explain a pathology, but hypotheses generated from statistical analyses of modeled structures open the door to better designed studies aiming to target specific regions in a 3D nucleus space which would not be predicted from one-dimensional data.

### Modeling to Enable New Wet-Lab Methods and Biological Insights on Chromatin Behavior

A powerful outcome of computational models of chromatin behavior is the opportunity to generate testable hypotheses on the physical properties of chromatin. A recent elegant example is the use of block copolymer models of chromatin to make predictions on the nature and dynamics of homotypic chromatin interactions occurring in microphase-separated compartment throughout the nucleus (Belaghzal et al., 2021). Testing these properties interestingly required further developments of the Hi-C methodology (which maps chromosomal interactions genome-wide) to accommodate a liquid chromatin phase (Belaghzal et al., 2021).

## Perspectives

### Considering Softness and Heterogeneity of the Nuclear Lamina

**FIGURE 5:**
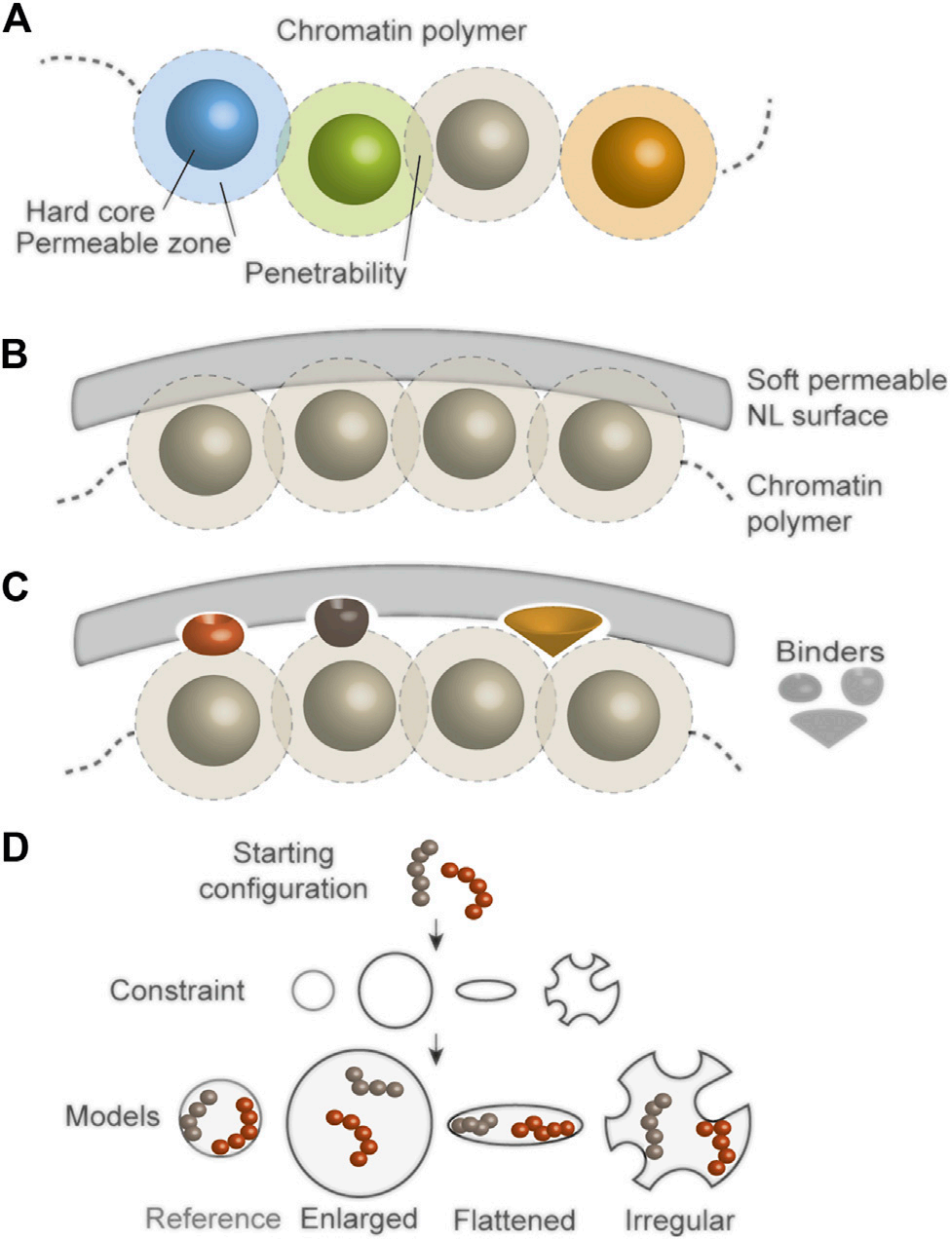
Perspectives on modeling chromatin-NL interactions **(A)** To enhance flexibility of a chromatin polymer, beads may be fitted with a permeable zone (limited by a hard core to prevent full bead clash) and an adjustable penetrability factor, to enable more realistic models of heterochromatin, and models of euchromatin interactions occurring as promoter-enhancer contacts (Siersbaek et al., 2017) or enhancer communities (Madsen et al., 2020) in transcriptionally active regions **(B)** A permeable zone can be similarly formulated within a NL surface to allow chromatin penetrance into the NL **(C)** Factors mediating chromatin anchoring with, or release from, the NL (binders) may also be introduced to refine chromatin-NL dynamics in biologically more relevant contexts than current models **(D)** Imposing nuclear envelope shape constraints in restraint-based genome models would allow predictions on 3D chromatin architecture in physiological contexts (e.g., flattened nuclei in adipocytes) or in pathological conditions (e.g., irregular nuclei in HGPS or cancer).

Despite their increasing complexity and power, chromatin models still ignore some information inherent to chromosome structure important for nuclear function, such as additional physical properties of chromatin. For example, chromatin domains can intermingle or partly invade the NL. So chromatin cannot simply be considered as a hard entity (non-penetrable beads in models). Instead, beads may be fitted with a soft permeable outer zone and an adjustable penetrability factor to enhance flexibility of the chromatin chain and better approximate chromosome compaction (Figure 5A). Similarly, a soft attraction potential may be introduced for interactions with a NL surface, a view justified by the void spaces penetrated by chromatin in the NL (Turgay et al., 2017). Thus, a NL surface should not necessarily be hard but be penetrable by polymer beads (Figure 5B).

Recent analyses of NL structure and organization (Nmezi et al., 2019; Turgay et al., 2017) suggest new options to improve models of chromatin-NL interactions. First, the distinct A- and B-type lamin networks of the NL, together with the identification of lamin A/C- and B-specific LADs (Forsberg et al., 2019) suggests that the strength of interaction potentials of chromatin with A- or B-type lamins could differentially be tuned. Second, the void volume of the NL could create space for binders mediating chromatin-NL interactions in block copolymer models, (Figure 5C). Binders, e.g., mimicking the lamin A-associated histone deacetylase HDAC3 (Demmerle et al., 2012), could also be introduced to simulate changes in chromatin states in LADs, such as those occurring in senescence (Chandra et al., 2012; Sadaie et al., 2013) or cancer (Dawson and Kouzarides, 2012). Other binders might control the release of chromatin from the NL during differentiation, senescence, or in disease.

### Modeling Interactions of the Nuclear Lamina With Euchromatin

Polymer models of chromatin folding at the NL in progeroid cells (Chiang et al., 2019) predict proximity of euchromatin to the NL only by tuning two parameters (see Figure 4C). This concept could be extended to model euchromatic and active LAD sub-domain, euchromatic regions dragged alongside heterochromatic LADs towards the NL, or associations of lamins with euchromatin in the nuclear interior. Locally fitting attraction potentials to these regions as a function of gene activity or epigenomic states should predict minimal conditions required to promote and maintain weaker NL-chromatin interactions. In this context, adding “binders” based on experimental evidence of factors involved in the modulation of chromatin-NL interactions, either as LADs or focal domains in LADs (e.g., CTCF) (van Schaik et al., 2021; Kaczmarczyk et al., 2022), should enable advances in our mechanistic understanding of these interactions. Modeling the behavior of a chromatin polymer with various thicknesses (Buckle et al., 2018) may also predict the dynamics of chromatin domains with varying compaction levels at the NL. By extension, it should also be possible to model lamin A interactions with euchromatin, which are modulated by histone acetylation (Ikegami et al., 2020).

### Introducing Nuclear Envelope Perturbations in 3D Genome Models

Further developments of 3D genome modeling to yield mechanistic insights into changes in nuclear architecture linked to disease would also be beneficial. Evidence implicates lamin mutations in alterations in LADs and chromatin conformation, and nucleus size and shape are also affected in cancer cells (de Leeuw et al., 2018). Introducing perturbations in the shape of the nucleus shell in restraint-based models may enable inference of unsuspected features of higher-order chromatin architecture linked to disease or differentiation (Katiyar et al., 2019; Dickinson et al., 2022) (Figure 5D). Such more advanced models could also in principle be supplemented with binders mediating chromatin interactions with nuclear bodies.

### Repetitive Elements in 3D Genome Models?

DNA repeats constitute more than half of the human genome and play a role in re-wiring epigenomes and gene expression programs in a variety of developmental and pathological conditions (Rebollo et al., 2012). Repeat elements are also frequently epigenetically altered in cancers (Dawson and Kouzarides, 2012). L1 elements (containing LINEs) are AT-rich, heterochromatic and enriched in LADs, whereas Alu repeats are more CG-rich and mainly found in euchromatic gene-rich A compartments. Accordingly, L1 and Alu repeats are relevant elements to consider in 3D genome models.

To our knowledge however, 3D models of chromatin have largely ignored DNA repeats because, 1) due to their repeat nature, repeat sequence reads are often discarded from analyses, 2) they are not relevant for the questions asked, or 3) they can simply be modeled as monomers (beads) alike any other chromatin domain. The latter is illustrated by the hidden inclusion of L1 repeats as LAD-associated topological domains (beads) in restraint-based whole-genome 3D models (Paulsen et al., 2017; Paulsen et al., 2018). Today however, repeats can in principle be explicitly included in 3D models as increasingly performant long-read sequencing technologies (De Bustos et al., 2016) and bioinformatics tools (Fernandes et al., 2020) provide more accurate estimates of their genomic localization. Their epigenetic states are also well characterized, and fluorescence *in situ* hybridization techniques allow their visualization in nuclei (Lu et al., 2021). Altogether, this information should be able to guide a phenomenological positioning of chromosome regions relative to a nuclear structure or boundary (e.g., peripheral positioning of L1-rich LADs) in simulation initiations, and validations of model predictions. It would also allow introduction of attraction potentials typical for heterochromatin (to cluster L1 elements) and of weaker potentials (to model Alu repeat aggregation). This view is supported by recent Hi-C and microscopy evidence of homotypic clustering of L1 and Alu repeats which compartmentalize the 3D genome (Falk et al., 2019; Lu et al., 2021).

### Enhancers and 3D Genome-Wide Association Studies

Recent evidence indicates that the NL constrains enhancers at the nuclear periphery, within LADs (Robson et al., 2017; Czapiewski et al., 2022; Madsen-Østerbye et al., 2022) and between LADs (Smith et al., 2021). By releasing these elements from the NL, disruption of NL-chromatin associations in laminopathies (McCord et al., 2013; Perovanovic et al., 2016; Paulsen et al., 2017) or cancer (Lenain et al., 2017) may alter the 3D interaction landscape of these elements in a manner reflected in genome-wide association studies (GWAS). GWAS typically link a genetic variant to a differentially expressed linearly proximal gene (an expression quantitative trait locus, or eQTL) using the nearest gene method. However, GWAS variants turn out to be located mainly outside genes, with only a minor fraction impacting nearby genes (Fadason et al., 2017; Mumbach et al., 2017; Fu et al., 2018). In a 3D space, variants likely affect more genes than projected and new eQTLs can be identified (Fadason et al., 2018; Buxton et al., 2019). Integration of 3D genomic perspectives, including LAD information, into GWAS studies may enhance identification of new genes and mechanisms underlying complex diseases, and in designing new treatments.

## Conclusion

Combinations of more sophisticated computational approaches with rapidly evolving wet-lab technologies such as high-throughput genome editing (Akhtar et al., 2013), live chromatin imaging (Germier et al., 2017), high-throughput fluorescence *in situ* hybridization (Finn et al., 2019) and biophysical techniques (Keizer et al., 2021), will expectedly lead to a clearer understanding of altered genome organization being a cause or consequence of disease.
